# Book and Pamphlet Notices

**Published:** 1859-08

**Authors:** 


### BOOK AND PAMPHLET NOTICES.

ANATOMY, DESCRIPTIVE AND SURGICAL. By HENRY GRAY, F. R. S., lecturer on Anatomy at St. George's Hospital. The Drawings by H. V. CARTER, M.D , late demonstrator of Anatomy at St. George's Hospital. The Dissections jointly by the author and Dr. Carter. With 363 engravings on wood. Philadelphia; Blanchard & Lea. 1859.

This is a republication by Blanchard & Lea, of a London book, on descriptive and surgical anatomy. The striking feature of the work is in the plan of illustrating the text.

In the cuts, which are numerous and of large size, the old fashioned system of numeration and foot references has been discarded, and in its stead has been substituted printing the name of each anatomical part upon the representation of the part itself. The figure thus explains itself. There are those who object to such plates, on the ground that it so simplifies the subject that it will prevent the student from prosecuting his anatomical studies upon that only reliable book the *cadaver*. But we do not think so.

We cannot but regard the method of illustrating adopted in this work as possessing many advantages to the student of anatomy.

Since the publication of illustrated works on anatomy and manuals, practical anatomy has actually increased in popularity. However, it is as essentially a diagramatic work, and its value will be estimated differently by different readers. Another feature of the work which should not be overlooked, is the application of human anatomy to practical surgery. We find the following in the preface:

"One of the chief objects of the author has been to induce the student to apply his anatomical knowledge to the more practical points in surgery, by introducing in small type, under each subdivision of the work such observations as show the necessity of an accurate knowledge of the part under examination."

The study of anatomy and surgery should go hand in hand; but we have no work in which the student can find the details of descriptive anatomy, combined at the same time with a concise account of the practical surgical relations of the parts.

The present volume is intended to supply this deficiency. The publishers have produced the American edition in an unexceptionable manner.

For sale by W. B. Keen, 146 Lake street, Chicago.

---

URINARY DEPOSITS, DIAGNOSIS, PATHOLOGY, AND THERAPEUTICAL INDICATIONS. By GOLDING BIRD, M.D., F.R.S. Edited by EDMUND LLOYD BIRKET, M.D., etc. A new American, from the fifth London edition. With 80 illustrations on wood. Philadelphia: Blanchard & Lea. 1859.

The profession, we think, have shown their appreciation of this work, by calling for five editions of it in London, and three in this country. The present volume, a new American, from the fifth London edition, has been revised and brought up to the present period. This work is one of very great practical value in investigating urinary diseases, and they are of such grave importance, that every physician ought to possess every possible aid in the diagnosis and treatment; and we know of no work which in so small a space will afford him so much material aid. The work is fully illustrated with finely executed wood cuts, and is every way creditable to the publisher.

For sale by W. B. Keen, 146 Lake street, Chicago.

---

A PAPER ON THE MANAGEMENT OF THE SHOULDERS IN EXAMINATION OF THE CHEST, INCLUDING A NEW PHYSICAL SIGN. Read before the New York Academy of Medicine. By JOHN W. CORSON, M.D., lecturer on diseases of the Chest, etc. (From the *New York Journal of Medicine* for March, 1859.) New York: H. Bailliere, 290 Broadway.

The object of this paper is to set forth the importance of the *position* of the shoulders during examination of the chest. The process in each case involves three principles—*thinning*, condensing and tightening; and is illustrated by the simple experiment of placing one fore-arm of a muscular man behind his back, while the other hangs loosely by his side, when the sound especially of percussion will be heightened below the clavicle of the stretched side in front.

Dr. Corson says, "from extensive trial, we firmly believe that, simple as they may seem, this management of the shoulders, these expedients for thinning, condensing and tightening the fleshy walls of the chest add fully one-third to our power of detecting the earliest signs of consumption."

---

CONTRIBUTIONS TO OPERATIVE SURGERY AND SURGICAL PATHOLOGY. By J. W. CARNOCHAN, Professor of Surgery in the New York Medical College; Surgeon in chief to the State Emigrant Hospital, etc. With illustrations drawn from nature. Philadelphia: Lindsay & Blackiston. 1859.

The above contributions are to be published in a series of ten numbers, to be issued quarterly. The second number of the series, containing 44 pages, of quarto size, and complete in itself, has been placed upon our table.

It contains a report of the following cases, with illustrations:

Case of Exsection of entire ulna.

Remarks on Neuralgia of the Face, with a case. Exsection of the trunk of the second branch of the fifth pair of nerves beyond the ganglion of Meckel, for severe neuralgia of the face; with three cases.

Terms of subscription, each number, 75 cents, to be paid for on delivery.

For sale at W. B. Keen's, 146 Lake street, Chicago.

---

FORTY-SECOND ANNUAL REPORT ON THE STATE OF THE ASYLUM FOR THE RELIEF OF PERSONS DEPRIVED OF THE USE OF THEIR REASON. Published by direction of Contributors. March, 1859. Philadelphia.

TRANSACTIONS OF THE STATE MEDICAL SOCIETY, OF MICHIGAN, at its Seventh Annual Meeting, held at Lansing, January 19th, 1859.

CONSTITUTION AND BY-LAWS OF THE CHICAGO ACADEMY OF MEDICAL SCIENCES. Adopted March, 1859.

---

A LECTURE ON IDIOCY. By JOHN M. GALT, M.D., Superintendent and Physician of the Eastern Lunatic Asylum of Virginia, at Williamsburg.

The title of this lecture may not, to some, give a promise of either much pleasure or profit in the perusal, as most men think idiots insusceptible of improvement by treatment, either physical, moral or intellectual. However, Dr. Galt thinks otherwise, and he wishes to show that, from the mere fact of institutions for this humane object being now multiplied over Europe, a great deal of scepticism which the subject encountered is removed. He notices a recent article in the *Medical Times and Gazette*, by Mr. Hutchinson, containing a report of the mode of procedure adopted in such cases at the hospice of Abendborg, under the care of a physician, who rejoices in the not very euphonic cognomen of Guggenbuhl. We presume the gentleman proceeds in the treatment on the supposition, shared by others, that in the great majority of his patients, their melancholy condition of mind depends on a torpor of its faculties, and that these only require awakening. This, at least, the details of treatment, which our space will not admit of, would lead us to infer. Certainly, Dr. Guggenbuhl has ample opportunities for observation and testing his practice, as there are, at least, 10,000 cretins, all idiots, in the Swiss Cantons; and he has, accordingly, profited by such, as his reputation is quite established in this department.

The connection, still ill-understood, between this state of mind and a residence in mountainous regions, so often insisted on by writers, is remarkable; but, like other pathological conditions of our nature, we infer this to be but one link in the chain of events antecedent to and productive of the affection under consideration.

We are told by Dr. Galt, that there are but 35,000 idiots in the United States—a fact, if well ascertained, consolatory, when we compare our lot in that respect with what we learn of Savoy, and the three departments of France—Isere, Basses, and Haute Alps, where, in the year 1850, no less than 54,000 of the inhabitants, or about 5 per cent. of the whole population, estimated at nearly a million, were idiots.

